# Relationships Among CEO Narcissism, Debt Financing and Firm Innovation Performance: Emotion Recognition Using Advanced Artificial Intelligence

**DOI:** 10.3389/fpsyg.2021.734777

**Published:** 2021-09-13

**Authors:** Lan Zhang, Biming Liang, Datian Bi, Yuan Zhou, Xiaohan Yu

**Affiliations:** ^1^School of Accounting, Jilin University of Finance and Economics, Changchun, Jilin, China; ^2^School of Management, Jilin University, Changchun, Jilin, China

**Keywords:** advanced artificial intelligence, emotion recognition, CEO narcissism, debt financing, innovation performance

## Abstract

Psychological research shows that as the main component of enterprise decision-making, CEOs are not completely rational, cognitive and psychological biases often influence their decision-making process. CEO narcissism has gradually attracted academic attention. Based on upper echelon theory and subconscious theory, this paper uses advanced artificial intelligence technology to quantify CEO narcissism as a kind of emotional intelligence. Taking A-share listed companies in China from 2010 to 2019 as research objects, this paper empirically tests the impact of CEO narcissism on debt financing and innovation performance. The results show that CEO narcissism has a significant positive impact on firm innovation performance. Debt financing plays a mediating role in the relationship between CEO narcissism and firm innovation performance. CEO narcissism can have a positive impact on firm innovation performance through debt financing. Compared with non-SOEs, SOEs' CEO narcissism has a more significant positive effect on debt financing and enterprise innovation performance. The research in this paper enriches psychology and organizational management and provides a reference for an enterprise's management decisions and for investors' investment decisions.

## Introduction

Corporate innovation is influenced by executives, especially narcissistic CEOs, such as Elon Musk, CEO of Tesla and SpaceX; Steve Jobs, former CEO of Apple; Bill Gates, former CEO of Microsoft; Mingzhu Dong, former CEO of Gree Air Conditioning Co.; Yueting Jia, former CEO of Letv; and other CEOs of famous enterprises, who showed different degrees of narcissistic personality tendencies. This phenomenon has attracted the attention of social groups and scholars to encourage enterprises to successfully break through technical barriers, improve their innovation performance and achieve success. According to the upper echelons theory, the management behavior of an enterprise is deeply influenced by the executive team (Hambrick and Mason, 1984; Hambrick, 2007). Executive traits, especially CEO traits (ability, risk-taking spirit, etc.), play a crucial role in a company's investment decision, especially in enterprise innovation (Demerjian et al., 2012; Sunder et al., 2017). According to the subconscious theory, there may be emotional structures that can be converted into consciousness in the subconscious, and the core of the subconscious is the representation of instinct (Freud, 1915). Narcissism belongs to the id and is the content of the subconscious, but it is manifested in some forms in the pre-consciousness, such as arrogance and arrogance. Experimental research on psychology shows that individual decision-making cannot be completely rational, and CEOs' psychological and personality characteristics will affect the business decisions and behavior of enterprises (Chatterjee and Poolock, 2017), especially playing a crucial role in enterprise innovation (Demerjian et al., 2012; Sunder et al., 2017). Existing studies mainly study the influence of executive traits on enterprise innovation from the perspectives of agency theory and branding theory, among which CEO narcissism is also gradually attracting academic attention (Chatterjee and Poolock, 2017). The influence of CEO narcissism on enterprise investment, financing, and innovation is gradually emerging, especially in the actual work and decision-making process, and the leadership traits formed by different CEOs' cognition will lead to different decision-making performance. However, there are relatively few studies on how CEO narcissism affects enterprise innovation performance, especially exactly how performance is affected. The current relevant studies mainly focus on CEO narcissism itself while ignoring the subjective initiative of narcissistic CEOs. In particular, the influence mechanism between the degree of CEO narcissism and enterprise innovation performance is less involved.

For enterprises, innovation will bring long-term benefits and achieve long-term development, but it is also full of uncertainty, high risk, long payback period, and other characteristics. The relevant literature has explored the factors that determine firm innovation, mainly involving the size of the firm, the structure of the market, and the characteristics of the industry (Shefer and Frenkel, 2005). However, these studies still fail to explain the phenomenon that enterprises with similar scale and external environment show great differences in innovation output. This is mainly because innovation activities are often accompanied by large capital and human resource investment, a high failure rate, and difficult to measure innovation achievements. Although existing studies have examined from different perspectives that debt financing is an important source of enterprises' access to innovation funds, they have neglected the effect of debt financing on innovation (David et al., 2008). The research of David et al. (2008) on Japanese enterprises found that the performance of enterprises that adjusted their debt structure with R&D investment activities was significantly better than that of enterprises that did not adjust their debt structure. Debt financing can provide financial support for enterprises' production, investment, innovation, research and development, and other activities and influence enterprises' innovation decision-making and efficiency. In the process of enterprise development, debt financing plays a pivotal role. Previous studies on debt financing mainly focus on corporate governance, corporate characteristics, and the external macro-environment. Kaplan et al. (2012) and Bernile et al. (2017) found that executives' decision-making styles have significant differences in the signals they send to the capital market. There are few studies on the role of debt financing based on CEOs' personal traits. As the decision makers of the company, the top management team, especially the CEO, play a key role in the major decisions of the company. Then, will CEO narcissism affect the innovation performance of enterprises? Will there be an impact on debt financing? What is the effect mechanism of debt financing on CEO narcissism and firm innovation performance? Based on the existing research and the connotation of CEO narcissism and subconscious theory, this study opens the black box between CEO narcissism and innovation performance and explores the impact of corporate debt financing on the relationship between CEO narcissism and enterprise innovation performance.

The possible contributions of this paper are as follows. First, advanced artificial intelligence technology is used to quantify CEO narcissism as a kind of emotional intelligence. Psychophysiological computing is a research method in emotional intelligence research that studies the relationship between human psychological activities and physiological changes through the analysis and calculation of physiological signals. The research of this paper organically combines the advanced artificial intelligence technology of psychology with the actual needs of organizational management, which is a bold innovation. Second, based on the theory of narcissism, connotation, and the subconscious, CEO narcissism affects the performance of enterprise innovation and its mechanism of action. In the past performance of enterprise innovation related literature from an agency theory perspective focused on the influence of the top management team for enterprise innovation decision-making, but paid less attention to the CEO personality effects on the performance of enterprise innovation. Third, this paper examines the impact of CEO narcissism on enterprise innovation performance from the perspective of debt financing and finds that the larger the scale of debt financing is, the more narcissistic CEOs will increase their investment in enterprise innovation and take more active initiative to improve innovation output and performance. This study enriches the literature in the fields of psychological theories and methods, CEO narcissism and corporate financing and provides a useful reference for current listed companies to optimize corporate governance, build a core team of senior executives, and improve enterprise innovation performance.

## Theoretical Analysis and Research Hypothesis

In recent years, narcissism has gradually become a focus of scholars. The study of narcissistic personality traits began in psychology and is regarded as a dark personality trait and a personality disorder. Since the 1980s, as psychologists and sociologists have conducted a series of scientific and experimental studies on the narcissistic personality, later scholars tend to regard the narcissistic personality as a relatively stable personality trait, which is not commendatory in itself (Wallace and Baumeister, 2002). In particular, with the development of the narcissistic personality inventory (NPI), the quantification of narcissistic personality traits has been realized. Since then, an increasing number of management scholars have incorporated it into their research field. Existing psychological studies have shown that personality has a significant impact on information processing and decision making, and individual decision making has difficulty achieving complete rationality. Narcissism is divided into explicit narcissism and implicit narcissism, but no matter what type of narcissism, the focus is on the self, desire, performance, and seeking the unique self.

Narcissism is a cognitive response (Lee et al., 2016). The main characteristics of narcissistic personality traits are as follows: First, narcissistic personality traits are relatively stable, basic, and deep-rooted (Campbell et al., 2004), usually endogenous, and less affected by external intervention and influence (Olsen, 2016). Second, narcissism is a combination of self-awareness and strong motivation, which affects external events from an internal perspective. Narcissists create a positive self-image by realizing their social status and self-worship. At the same time, narcissists have motivations such as yearning for their own rights and craving for the affirmation of others. Finally, Chatterjee and Hambrick (2007) pointed out that narcissistic personality traits include three important components: cognition, motivation, and behavior strategy. In terms of cognition, narcissistic CEOs have extremely inflated self-concepts and believe that they have privileges. They are always self-centered, believing that their decisions are always right and resisting others' disagreements (Campbell et al., 2011). In terms of motivation, narcissistic CEOs strongly pursue the power to control others and the praise of others to maintain their inflated self-concept (Chatterjee and Poolock, 2017). In terms of behavior, narcissistic CEOs usually adopt two strategies of “self-improvement” and “self-defense” to achieve their pursuit of power and the praise of others. On this basis, this paper summarizes the narcissistic personality characteristics as follows: narcissists are gifted, have extraordinary intelligence, and attraction, are good at sketching grand visions, and are very eager to exercise power. These characteristics urge narcissists to constantly seek recognition, affirmation and praise to maintain their sense of superiority.

### The Impact of CEO Narcissism on Debt Financing

CEO narcissism often underestimates risk and overestimates earnings, leading to more radical risk taking. An “above average” bias in judging and making decisions by narcissistic CEOs (Larwood and Whittaker, 1977; Alicke, 1985) is mainly characterized by two aspects: (1) It is easy to overestimate the possibility of success of an event while ignoring its risk. (2) It is easy to attribute success to one's own ability and attribute failure to poor luck (Miller and Ross, 1975). CEO narcissism has an important impact on corporate financing strategy and capital structure. In recent years, scholars have carried out a series of theoretical and empirical studies on the impact of CEO narcissism on corporate financing preference and achieved certain results. Shefrin and Statman (1999) argues that narcissistic CEOs overestimate the company's future earnings which they do not want to share with new shareholders; they prefer to issue bonds rather than stocks when choosing means of financing. Heaton (2002) confirmed the pecking order financing theory from the perspective of over-optimism caused by CEO narcissism. Overly narcissistic CEOs will think that a company's value is underestimated by the market. If there is not sufficient cash, even if there are investment projects with positive net present value, CEOs will refuse because of the high financing cost. If companies have to choose external financing, CEOs will also prefer debt financing because stock prices are more sensitive to market expectations. Therefore, the financing order of narcissistic CEOs is endogenous financing-debt financing-equity financing. Hackbarth (2008) study shows that narcissistic CEOs overestimate the profitability of investment projects and the ability of enterprises to reduce risks and use higher debt ratios to issue debt more frequently, especially short-term debt. Hackbarth's model shows that narcissistic CEOs believe that their companies and investment projects can recover cash flow as soon as possible. Hackbarth also found that debt financing could raise corporate value in two ways: first, by limiting CEOs' arbitrary transfer of funds to reduce agency costs; second, by reducing conflicts between shareholders and creditors. The empirical study of Malmendier and Tate (2005) found that narcissistic CEOs are more cautious about external financing than ordinary leaders. When overseeing external financing, they are willing to choose more debt financing and less stock issuance. In addition, external institutions, especially the news media, tend to pay more attention to the risky decision-making of enterprises, and debt financing for R&D investment is a risky behavior that can bring more attention to enterprises and to some extent can satisfy the narcissistic CEO's desire for external attention and increase their sense of superiority. Based on these observations, the following assumptions are made:

H1: CEO narcissism is positively correlated with debt financing.

### The Impact of CEO Narcissism on Corporate Innovation Performance

Innovation is the driving force of enterprise growth, and R&D investment can guarantee innovation results. The sunk costs, opportunity costs, and inherent risks of R&D investment once made many CEOs unwilling to invest more in R&D innovation. However, narcissistic CEOs are different. Driven by desire and achievement motivation, narcissistic CEOs' behavior has an obvious risk seeking tendency. In strategic decision-making, narcissistic CEOs are more inclined to “high risk and high yield” schemes. To seek opportunities to show their superiority, narcissistic CEOs are more willing to adopt cutting-edge technology. Overconfidence makes narcissistic CEOs overestimate returns, underestimate risks, and think that the probability of success of R&D innovation is greater (Campbell et al., 2004). Gerstner et al. (2013) found that narcissistic CEOs tend to adopt cutting-edge technology to obtain the stakeholders' general appreciation. Kumar (2019) points out that narcissistic leaders tend to be bold. In order to meet their own needs, narcissistic leaders often take risks and accept challenges, and they have strong internal driving forces. Therefore, they will positively affect employees' innovative behavior and inject fresh vitality into organizational change. The characteristics and difficulties of innovation projects can often attract the attention of narcissistic CEOs. On the one hand, with high uncertainty, the risk of innovation projects is greater and more challenging. Once successful, it can often demonstrate the talents of CEOs, thus attracting more attention and praise. On the other hand, the success of innovation projects can often become a key breakthrough in the growth momentum of enterprises, increase the scientific and technological content and added value of products, accelerate the process of enterprise innovation and improve enterprise value. Therefore, narcissistic CEOs will increase the intensity of innovation investment.

Based on this motivation, narcissistic CEOs with their own capabilities will adopt a series of corporate decisions to promote the implementation of innovation projects and the improvement of innovation performance. CEO narcissism will pay more attention to innovation projects (Hirshleifer et al., 2012). Rosenthal and Pittinsky (2006) found in relevant studies that narcissistic CEOs will work harder than ordinary CEOs to maintain their sense of superiority. Narcissistic CEOs can significantly promote the innovation performance of knowledge workers and help to create a cultural atmosphere of innovation in enterprises to promote the innovation output performance of enterprises. Compared with non-narcissistic CEOs, narcissistic CEOs can better identify valuable risk projects (Gervais et al., 2011) to obtain innovation results. To better promote the improvement of enterprise innovation performance, narcissistic CEOs tend to modify the enterprise strategy, prompting enterprises to adopt more science and technology strategies (Galasso and Simcoe, 2011) so that the enterprise goals, profit model, budget management, organizational structure, and daily operation of departments are closely related to the science and technology strategy and provide comprehensive cooperation for the implementation of innovation projects. Narcissistic CEOs also tend to recruit more innovative employees (Steen, 2005), which is conducive to the team's efforts toward common goals and missions. The degree of organizational identity and organizational consistency is higher, which can guarantee the smooth implementation of innovative projects. Based on this, the following assumptions are made:

H2: CEO narcissism is positively correlated with corporate innovation performance.

### The Mediating Role of Debt Financing on the Relationship Between CEO Narcissism and Corporate Innovation Performance

Enterprise innovation activity is a high-risk decision-making behavior. The initial stage of innovation activity requires a large amount of capital investment and the cooperation of various resources. The process is full of uncertainties. It may not be profitable for many years, or it may end in failure (Ham et al., 2018). Therefore, a CEO who can make innovation investment decisions must have certain power, dare to take risks, and have enough courage and the ability to take risks, and be willing to invest a large amount of debt financing in innovation and research of the enterprise. Narcissistic type CEOs have these characteristics. For example, Buyl and Boone (2017) found a positive correlation between CEO narcissism and corporate risk-taking. Because narcissistic CEOs underestimate the risk of failure of innovative projects, when the company is in a situation of high debt financing, they often adopt aggressive R&D investment strategies. The behavior of an enterprise to maintain its reputation and brand through innovation investment will be regarded by creditors as a signal for the enterprise to initiate active competitive projects. It is easy to obtain the approval of creditors and can reduce the company's financing costs. Narcissistic CEOs strongly pursue power and honor, and this vote of confidence will undoubtedly satisfy the psychological needs of narcissistic CEOs. At the same time, the information asymmetry, and asset substitution problems caused by external financing will also encourage narcissistic CEOs to engage in risk-taking behaviors and increase investment in innovation. On the other hand, under the constraints of financing, companies will strive to improve the efficiency of R&D and innovation. Based on the positive effects of narcissistic CEOs and corporate innovation performance described in the previous article, corporate innovation performance may be improved.

Based on the above analysis, in this article, it is proposed that the improvement in corporate innovation performance is the joint result of CEO narcissism and the increase in debt financing. CEOs with narcissistic characteristics can better optimize the allocation of resources invested and guarantee realization of the R&D investment from all aspects. With increased efficiency, CEO narcissism results in improved innovation performance. Narcissistic CEOs overestimate the profitability of companies or investment projects. They prefer lower-cost but higher-risk financing methods and debt financing, especially short-term debt financing. Debt financing also affects corporate innovation decisions through constraints and incentives on corporate managers. Narcissistic CEOs are more adventurous, and adventurous managers can alleviate the problem of underinvestment, which is conducive to enhancing corporate value (Thakor and Goel, 2008). Narcissistic CEOs are often more creative and can discover more opportunities for innovation. To better promote the improvement of corporate innovation performance, narcissistic CEOs will proactively acquire and integrate the information and resources needed for innovation, and by modifying corporate strategies, they will encourage companies to adopt more technology strategies. According to the paradigm of psychology, debt financing acts as a bridge, and facilitator in the relationship between CEO narcissism and corporate innovation performance based on the psychological characteristics of narcissism. Leaders' irrationality is transmitted to the company through the financing decisions they make and then affects the company's performance. Therefore, debt financing plays an intermediary role in the influence of CEO narcissism on the company's innovation performance. Based on this, the following hypothesis proposed:

H3: Debt financing plays a mediating role in the relationship between CEO narcissism and corporate innovation performance.

### The Moderating Effect of the Nature of Enterprise Ownership

State-owned enterprises and non-state-owned enterprises have different structures, and the nature of corporate ownership may have different effects. This article speculates that state-owned enterprises have a significant positive moderating effect on the relationship between CEO narcissism, debt financing and corporate innovation performance. Based on the theory of resource dependence, state-owned enterprises are an important subject of national independent innovation and an indispensable part of the national innovation system. Compared with non-state-owned enterprises, state-owned enterprises shoulder more important social responsibilities to promote the high-quality development of the national economy through technological innovation. At the same time, state-owned ownership is regarded as a resource-rich external shareholder that can provide reliable resource support for enterprise technological innovation. State-owned enterprises have concentrated national high-quality R&D resources and possess strong innovation capabilities. They are the backbone of my country's innovation-driven development. CEOs with a high degree of narcissism will make full use of national innovation resources, carry out debt financing, increase R&D investment, and improve corporate innovation performance. State-owned enterprises can reduce the uncertain risks caused by system and policy changes and the uncertainty of the external environment, thereby promoting enterprise innovation (Choi et al., 2011). In recent years, the Chinese government has placed technological innovation at the core of national development and has made a series of major decisions and deployments for the implementation of an innovation-driven development strategy. Based on this, the narcissistic CEOs of state-owned enterprises actively cater to national policies to demonstrate their image as excellent leaders and then increase debt financing to promote R&D investment and improve corporate innovation performance. In addition, to obtain promotion and attention from the outside world, compared with non-state-owned enterprises, narcissistic CEOs of state-owned enterprises pay more attention to the improvement of the company's innovation performance. Innovation performance has become an important criterion for measuring the ability of enterprise managers, and it is also an important part of the promotion and assessment of state-owned enterprise managers. Narcissistic CEOs are more energetic and confident, make decisions more decisively and are more inclined to increase debt financing to increase corporate R&D investment to establish their image as a “visionary.” Therefore, the following hypotheses are put forward:

H4a: Compared with non-state-owned enterprises, the CEO narcissism of state-owned enterprises has a more significant impact on debt financing.

H4b: Compared with non-state-owned enterprises, the CEO narcissism of state-owned enterprises has a more significant impact on innovation performance.

**Figure 1:**
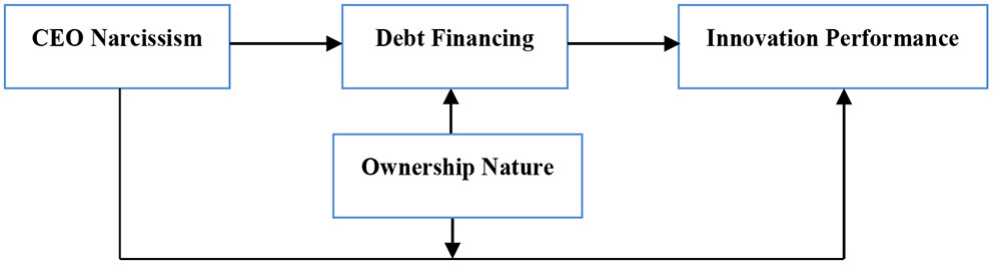
Research framework.

Based on the above analysis, the research framework constructed in this paper is shown in Figure 1.

## Research Design

### Variable Definition and Measurement

#### CEO Narcissism

Researchers that studied artificial intelligence incorporated the theory of multiple intelligences and the concept of emotional intelligence; thus, a new direction of cognitive intelligence and emotional intelligence emerged. Emotional intelligence is based on the existing technology of artificial intelligence, and on this basis, it enhances the ability of artificial intelligence to understand emotion, generate emotion, and express emotion to construct human-like intelligence with emotion and empathy. The introduction of emotional intelligence makes artificial intelligence not only intelligent but also emotional. Emotional intelligence is a machine that finds and identifies human emotions by analyzing users' facial expressions, physiological signals, eye movements, voices, and content released on social media. It understands emotions, generates empathy, and expresses emotions. Emotional intelligence can be applied to medical treatment, education, production, commerce, entertainment, and other fields by giving machines emotion to achieve harmonious human-computer interaction. When quantifying CEO narcissism, this paper applies the new discovery of emotional intelligence to quantify the degree of CEO narcissism by identifying the content released by CEOs on social media.

CEO narcissism (Ceonar) refers to the growing self-consciousness of CEOs and their focus on self-concept (Campbell et al., 2011). At present, the most commonly used method to measure the degree of individual narcissism is the Narcissism Personality Inventory (NPI). However, CEOs of listed companies are usually not willing to take the sensitivity test of individual personality traits. Even if some CEOs are willing to take the test, the test results will also be affected by subjective biases caused by social expectations. Therefore, based on the study of Olsen et al. (2014) and Chatterjee and Hambrick (2011), this paper selects objective measurement indicators according to the four narcissism dimensions proposed by Emmons and Robert (1987). Combined with China's national conditions, individual indicators are excluded, and new indicators are added. Finally, four objective measurement indicators are used to measure the degree of CEO narcissism. The relationship between the selected indicators and the four sub-dimensions of CEO narcissism is shown in Table 1.

**Table 1:** The relationship between the selected indicators and the four sub-dimensions of CEO narcissism.

**Four subdimensions of narcissism (Emmons and Robert**, **1987****)**				
	**Leadership/authority**	**Arrogance/self-worship**	**Superiority/arrogance**	**Utilitarian/power desire**
Typical items in Narcissistic Personality Inventory (NPI)	People always recognize my authority. I was born a leader	I often admire myself in the mirror. I am an excellent person.	I love being the focus of attention. I like to be above others	I must get others awe and respect. I envy others' achievements
**The correspondence between indicator meaning and narcissism subdimensions**				
Indicators selected in this article				
Proportion of CEO news reports on company home page (sub1)	I am the core of the company.	I like the high attention CEO brings me		My site, I 'm the owner, I 'm the company news
The proportion of CEOs using the first person singular (sub2)	Corporate performance is my own achievement, not my team		I am the company, the company is me	My importance should be highlighted.
Proportion of original social media works (sub3)		My point is right. I am the embodiment of truth.	I am the example of others, they should imitate me, follow my steps	

To further test the consistency of the four objective indicators, A-mos21.0 was used for confirmatory factor analysis (CFI = 0.91, EFI = 0.88, NNFI = 0.87, RMSEA = 0.08) and reliability analysis. The Cronbach's α value of the measurement item is 0.68, which is higher than the minimum acceptable value of 0.65, indicating that the CEO narcissism measurement index has good reliability and validity. Based on the above tests, the average value of the four indicators is finally standardized as the CEO narcissism index.

#### Debt Financing

Debt financing (Debt) represents the scale of corporate debt financing, which is the intermediary variable of this study. As China's current financial system and banking system are relatively stable, banks remain dominant in corporate external financing. In this paper, the total amount of bank loan, namely the balance of long-term loan and short-term loan, is used to measure debt financing. The total amount of bank loan reflects the total ability of enterprises to borrow (Lu et al., 2012). So in light of Kim and Lee (2008) and other researchers, the ratio of bank loans to total corporate assets is used to measure the scale of corporate debt financing, which is the sum of long-term and short-term borrowings.

#### Innovation Performance

The dependent variable innovation performance (IP) includes innovation input and innovation output. Innovation input is measured by R&D expenditure/operating income, and innovation output is measured by the number of patent applications. Enterprises generally apply for patents in the year when they invent new technologies, and the year of patent authorization may lag behind the year of patent application. In addition, compared with patents, the value of new products must be tempered by the fact that enterprises do not apply for patents anonymously; thus, patent applications can measure innovation output more comprehensively, and can better reflect the return of enterprise innovation by reflecting the degree of market acceptance of new products (Wang and Kafouros, 2009). Therefore, it is more objective to measure innovation output performance by the number of patent applications. The greater the number of invention patent applications is, the higher the innovation performance of enterprises is.

#### Nature of Ownership

Ownership nature, according to the nature of the actual controller, is divided into state-owned (SOE) and non-state-owned enterprises, state-owned enterprises for 1, and non-state-owned enterprises for 0.

#### Control Variables

Drawing on previous studies on CEO narcissism, debt financing and innovation performance, control variables are mainly introduced at the enterprise level, corporate governance level and other levels. The control variables at the firm level include firm size (Size) and firm return on total assets (Roa). The control variables of corporate governance include CEO age (Ceoage), CEO education (Education), board size (Bsize) and the proportion of independent directors (Indir). The above variables and explanations are shown in Table 2.

**Table 2:** Definition and measurement of variables.

**Variable type**	**Variable name**	**Symbol**	**Measurement method**
Dependent variable	Innovation performance	IP	Number of patent applications
Explanatory variable	CEO narcissism	Ceonar	CEO narcissism index
Mediating variable	Debt financing	Debt	(Short-term loans + long-term loans) / total assets
Moderating variable	Nature of ownership	SOE	dummy variable, state-owned enterprise value 1, non-state-owned enterprise value 0
Control variables	Enterprise size	Size	Ln (Total assets)
	Return on total assets	Roa	Net profit / total assets
	CEO age	Ceoage	Actual age of the year (age)
	CEO degree	Education	≤ specialist 1, undergraduate 2, master 3, doctor 4
	Number of board of directors	Bsize	Number of formal members of the board of directors
	Proportion of independent directors	Indir	Proportion of independent directors

### Model Construction

To test the relationships among CEO narcissism, corporate debt financing and corporate innovation performance, this paper establishes the following equations:


(1) $$\begin{array}{l} \text{Debt}=\phi_{0}+\phi_{1}\text{Ceonar}+\phi_{2}\text{Size}+\phi_{3}\text{Roa}+\phi_{4}\text{Ceoage}\\ \;+\phi_{5}\text{Education}+\phi_{6}\text{Bsize}+\phi_{7}\text{Indir}+\varepsilon \end{array}$$

(2) $$\begin{array}{l} \text{IP}=\beta_{0}+\beta_{1}\text{Ceonar}+\beta_{2}\text{Size}+\beta_{3}\text{Roa}+\beta_{4}\text{Ceoage}\\ \;+\beta_{5}\text{Education}+\beta_{6}\text{Bsize}+\beta_{7}\text{Indir}+\mu \end{array}$$

(3) $$\begin{array}{l} \text{IP}=\lambda_{0}+\lambda_{1}\text{Ceonar}+\lambda_{2}\text{Debt}+\lambda_{3}\text{Size}+\lambda_{4}\text{Roa}\\ \;+\lambda_{5}\text{Ceoage}+\lambda_{6}\text{Education}+\lambda_{7}\text{Bsize}+\lambda_{8}\text{Indir}\\ \;+\sigma \end{array}$$

Model (1) tests the relationship between CEO narcissism and debt financing; if ϕ_1_ is significantly positive, then it confirms hypothesis H1. Through model (2) testing the relationship between CEO narcissism and enterprise innovation performance, if β_1_ is significantly positive, then hypothesis H2 is confirmed. Model (3) tests the mediating effect of debt financing on the relationship between CEO narcissism and enterprise innovation performance. To test the moderating effect of the nature of enterprise ownership, this paper uses the above three models to test the grouping of state-owned enterprises and non-state-owned enterprises.

### Sample Selection

This paper selects 2010–2019 Shanghai and Shenzhen A-share listed companies as the initial research sample. Sample selection follows the following principles: (1) Financial and insurance listed companies are excluded. (2) The listed companies whose transaction statuses are ST and ^*^ST are excluded. (3) Companies whose listing year is <3 years or whose CEO is <3 years are excluded. (4) Incomplete financial indicator data on CEO narcissism, debt financing, innovation performance or missing listed companies are excluded. After the above steps, a total of 402 sample enterprises and a total of 2,332 observations constitute the company-year data sample; among them, 103 state-owned enterprises, a total of 598 sample points and 299 non-state-owned enterprises, a total of 1,734 sample points. CEO narcissism data are obtained through a large amount of text mining through financial websites, Baidu search engines, microblog and blog network communication platforms, and corporate homepage news. The sample enterprise financial data are obtained from the CSMAR and Wind databases.

## Empirical Research

### Descriptive Statistics

Table 3 shows the descriptive statistical results of the relevant variables. As shown in Table 3, the minimum value of innovation performance (IP) is 0, the maximum value is 824, and the standard deviation is 118.91, indicating that different enterprises have substantial differences in innovation performance. The minimum value of the CEO narcissism index (Ceonar) is 0.020, and the maximum value is 0.589. The mean value is 0.112, to a certain extent, indicating that the degree of narcissism of CEOs varies greatly, and the majority of people have a low degree of narcissism. The minimum value of debt financing (Debt) is 0, and the maximum value is more than 50%. There is a large difference between the two values. The average difference is 0.181, which indicates that the total debt financing of listed companies in China mainly consists of bank loans that amount to more than 18%. The standard deviation is 0.146, which indicates that the debt financing scale obtained by different enterprises differs greatly. The descriptive statistical analysis of other variables is shown in Table 3 and will not be repeated.

**Table 3:** Descriptive statistical results of variables.

**The variable name**	**Mean value**	**Standard deviation**	**Minimum value**	**Maximum value**
IP	45.73	118.91	0.00	824.00
Ceonar	0.112	0.208	0.020	0.589
Debt	0.181	0.146	0.000	0.569
Size	24.126	1.351	19.425	26.536
Roa	0.052	0.063	−0.183	0.192
SOE	0.412	0.498	0.000	1.000
Ceoage	46.892	5.726	30.000	68.000
Education	3.655	0.729	1	4
Bsize	8.725	1.826	5.000	15.000
Indir	0.384	0.061	0.095	0.800

### Correlation Analysis

Table 4 carries out Pearson test on the correlation of main variables. The results show that CEO narcissism, debt financing and innovation performance pass Pearson correlation test. In addition, the absolute value of the maximum pairwise correlation coefficient between variables is 0.379, indicating that there is no serious multicollinearity between variables.

**Table 4:** Correlation analysis results.

**变量**	**1**	**2**	**3**	**4**	**5**	**6**	**7**	**8**	**9**	**10**
1. IP	1									
2. Ceo_Nar	0.065^**^	1								
3. Debt	0.084^***^	0.112^***^	1							
4. Size	0.379^***^	0.181^**^	0.198^**^	1						
5. Roa	−0.051^*^	−0.034	−0.177^**^	0.336^***^	1					
6. SOE	0.127^***^	0.265^**^	0.063^**^	0.246^**^	0.017^*^	1				
7. Ceoage	0.037	−0.128^**^	0.092^*^	0.037	0.014^*^	−0.019	1			
8. Education	0.122^***^	0.009	−0.005^**^	−0.017^***^	0.088^*^	0.004	−0.012	1		
9. Bsize	0.021^*^	0.018	0.155^**^	−0.002^**^	0.013^*^	−0.034	0.018	0.011	1	
10. Indir	0.256	0.023^*^	−0.009	−0.025	−0.030	0.041^*^	0.072	0.071^*^	0.056	1

* *p < 0.1*,

** *p < 0.05*,

*** *p < 0.01*.

### Hypothesis Testing

#### Main Effect Test

This paper uses regression analysis. First, the impact of CEO narcissism on debt financing is analyzed. Second, the influence of CEO narcissism on innovation performance is investigated. Finally, the mediating effect of debt financing on the relationship between CEO narcissism and enterprise innovation performance is tested in the next part. The results are shown in Table 5.

**Table 5:** Regression analysis results.

**Variables**		**The dependent variable**		**The dependent variable**		
		**(Debt)**		**(IP)**		
		**Model 1**	**Model 2**	**Model 3**	**Model 4**	**Model 5**
Control variables	Size	0.081 (74.32)	0.313^***^ (5.24)	−0.230^***^ (−3.78)	−0.155^***^ (−2.36)	−0.117^**^ (−1.92)
	Roa	0.126^**^ 2.11	0.123^**^ (2.01)	0.002 (3.21)	0.003 (3.12)	0.002 (3.19)
	Ceoage	0.142^**^ (3.95)	0.136^**^ (3.62)	−0.021^*^ (−2.31)	−0.016^*^ (−1.68)	−0.013^*^ (−1.78)
	Education	0.026^*^ (1.15)	0.017^*^ (1.11)	0.152^**^ (2.78)	0.191^***^ (3.12)	0.175^***^ (2.78)
	Bsize	0.002^***^ (3.19)	0.0014^*^ (2.25)	0.098^***^ (1.67)	0.084^***^ (1.72)	0.080^***^ (1.21)
	Indir	0.051^*^ (3.69)	0.005 (3.35)	0.51 (1.745)	0.63 (1.911)	0.57 (3.79)
	Year	Control	Control	Control	Control	Control
	Industy	Control	Control	Control	Control	Control
The independent variable	Ceonar		0.282^***^ (5.26)		0.166^**^ (2.58)	0.096^*^ (1.58)
Intervening variable	Debt					0.134^*^ (2.16)
Adjusted R2		0.501	0.338	0.847	0.470	0.261
F		405.2	206.46	86.65^***^	48.892^***^	10.25^***^

* *p < 0.1*,

** *p < 0.05*,

*** *p < 0.01*.

Model (1) and Model (2) examine the relationship between CEO narcissism and debt financing. Model (1) is an estimate containing only control variables, Model (2) takes CEO narcissism as the explanatory variable and debt financing as the explained variable. The results show that H1 passes the test (β = 0.282, *P* < 0.001). Model (3) and Model (4) examine the relationship between CEO narcissism and innovation performance. Model (3) is an estimated result containing only control variables, Model (4) takes CEO narcissism as the explanatory variable and innovation performance as the explained variable. The results show that H2 is tested (β = 0.166, *p* < 0.005).

#### Mediating Effect Test

For the mediating effect, this paper will use the 3 steps described by Baron et al. to verify its significance and use the Sobel, Aroian, and Goodman tests to further verify its significance. According to Baron (1986), there are three regression steps for mediator variables: step 1 is the measurement of the relationship between the predictor variable and the dependent variable, and its β value should be significant. Step 2 is the measurement of the relationship between the independent variable and intermediate variable, and its β value should also be significant. Step 3 considers independent variables and intermediary variables simultaneously and measures the relationship between them and dependent variables. At this point, the β value between the independent variable and the dependent variable is lower than the β value in Step 1, and it is completely true for those that are not significant, while it is partially true for those that are significant. However, the relationship between the mediator variable and the dependent variable is still significant, as shown in Table 6.

**Table 6:** Measurement steps of mediating variables.

**Steps**			**β value**	**Establishment condition**
Step1	The independent variable	The dependent variable	β1	β1 should be significant
Step2	The independent variable	Intervening variable	β2	β2 should be significant
	The independent variable	The dependent variable	β3	β4 should be significant, β1>β3
Step3	Mediating variable	The dependent variable	β4	β3 is completely true without significance: The significance is partially true

This paper uses the mediated regression method to study the relationship between CEO narcissism, debt financing and innovation performance. The specific test results are shown in Table 7.

**Table 7:** Mediating effect test results.

**Step**	**Explanatory variable**	**Explained variable**	**β value**	**Hypothesis condition**
Step1	The independent variable	The dependent variable	β1	β1 should be significant
	Ceonar	IP	0.166^**^	significant
Step2	The independent variable	Mediating variable	β2	β2 should be significant
	Ceonar	Debt	0.282^***^	significant
Step3	The independent variable	The dependent variable	β3	β3 should be significant
	Ceona	IP	0.096^*^	significant,0.166>0.096,partial mediating effect holds
	Mediating variable		β4	β4 should be significant
	Debt		0.134^*^	significant

* *p < 0.1*,

** *p < 0.05*,

*** *p < 0.01*.

**Figure 2:**
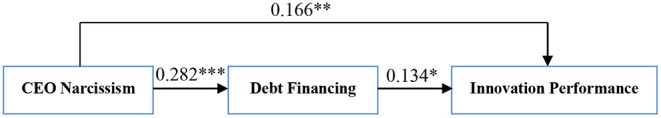
Debt financing mediating effect results. *p < 0.1, **p < 0.05, and ***p < 0.01.

The results in Table 7 show that both Step 1 and Step 2 meet the conditions. In Step 3, the partial mediating effect of debt financing on the relationship between CEO narcissism and innovation performance is established, since 0.166 > 0.096 meets the principle of partial mediating effect described by Baron et al. Therefore, H3 is partially supported. Figure 2 shows the mediating effect results of debt financing.

To further verify the mediating effect, the Sobel method and the bootstrap method were selected for retesting, Table 8 shows the mediating effect test results. These two methods could reduce the impact of the second type of error on the research results. Bootstrapping is currently the most ideal, and the latest inspection intermediary effect method has been applied in different disciplines. The method of samples takes back the whole sampling method for repeated sampling. Each time, the parameters obtained from the sample average are the final results, have high effectiveness in statistical results, and provides more reliable results. If the Sobel test results show a significant Z value, the confidence interval of the bootstrap test results does not contain 0, indicating that the mediation test has been passed. Table 6 shows the results of the Sobel test and bootstrap test. The Z-value is 1.69, which is significant at the level of 1%, passing the Sobel test. The confidence interval of the bootstrap test is 0.0005 to 0.002, excluding 0, and the result is robust after the test.

**Table 8:** Mediating effect test results.

**Sobel test**	**Bootstrap test (95% confidence interval)**	
Z value	The lower limit	Ceiling
1.69^***^	0.0005	0.002

*** *p < 0.01*.

#### Test of the Moderating Effect of the Nature of Enterprise Ownership

According to the nature of ownership, the samples are divided into state-owned enterprises and non-state-owned enterprises for regression. Table 9 shows the regression results. The results of Model 1 and Model 2 show that in SOEs, CEO narcissism is significantly positively correlated with debt financing. Compared with non-SOEs, CEO narcissism in SOEs has a greater impact on debt financing (0.385 > 0.164). H4a is verified. The results of Models 3 and 4 showed that CEO narcissism is significantly positively correlated with innovation performance. Compared with non-state-owned enterprises, the CEO narcissism of state-owned enterprises has a greater impact on innovation performance (0.284 > 0.133). H4b is verified.

**Table 9:** Moderating effect test results.

**Variable**	**Debt**		**IP**	
	**Model 1**	**Model 2**	**Model 3**	**Model 4**
	**State-owned**	**Non-state**	**State-owned**	**Non-state**
Size	0.286^***^ (4.79)	0.178^***^ (2.95)	−0.174^***^ (−2.19)	−0.109^*^ (−1.76)
Roa	0.214^*^ (3.47)	0.112^*^ (1.86)	0.005 (1.12)	0.001 (1.03)
Ceoage	0.125^*^ (1.96)	0.347^*^ (1.978)	−0.081^*^ (−1.28)	−0.098^*^ (−1.69)
Education	0.013^*^ (0.522)	0.096^**^ (1.575)	0.265^***^ (4.34)	0.123^*^ (2.12)
Bsize	0.107 (1.75)	0.031^*^ (0.513)	0.064^*^ (1.01)	0.104^***^ (1.79)
Indir	−0.026 (−0.29)	0.013 (0.211)	0.251 (3.97)	0.302 (4.19)
Year	Control	Control	Control	Control
Industy	Control	Control	Control	Control
Ceonar	0.385^***^ (5.26)	0.164^***^ (2.581)	0.284^**^ (4.76)	0.133^**^ (2.12)

* *p < 0.1*,

** *p < 0.05*,

*** *p < 0.01*.

#### Robustness Test

To ensure the reliability of the research conclusions, we carried out a series of robustness tests. For the CEO signature size data used in this paper, the approach of Ham et al. (2017, 2018) was used to measure the degree of narcissism of CEOs. The greater the value is, the higher the degree of narcissism of the CEO. IPO prospectuses and prospectuses^1^ of listed companies from 2010 to 2019 from Juchao Information Network, including 2,663 prospectuses and 1,043 prospectuses, a total of 3,706 documents^2^ were downloaded. Then, Python deep learning software was used to collect the CEO signature size. The specific steps are as follows: First, the CEO name coordinates are manually determined. The program then returns the number of pixels that record the size of the signature and the area it occupies. Then, the data are standardized to obtain the area of the signature. Finally, the data obtained by dividing the signature area by the number of words in the name were taken as the proxy variable of CEO narcissism, which was substituted into the model for regression analysis, and the research conclusion remained unchanged.

## Conclusion and Limitation

### Research Conclusion

Although existing studies have explored the impact of CEO narcissism on enterprise innovation performance from different perspectives, the research is not perfect, and there are few studies from the perspective of debt financing. Based on the sample of A-share listed companies in China from 2010 to 2019, this paper incorporates CEO narcissism, corporate innovation performance and debt financing into a framework for analysis. Debt financing is used as an intermediary variable to test its role in the relationship between CEO narcissism and corporate innovation performance. The empirical results show that CEO narcissism is positively correlated with debt financing. The higher the degree of CEO narcissism is, the larger the scale of debt financing is. CEO narcissism is positively correlated with enterprise innovation performance. The higher the degree of CEO narcissism is, the higher the enterprise innovation performance is. Debt financing plays a mediating role between CEO narcissism and corporate innovation performance. Compared with non-state-owned enterprises, CEO narcissism has a more significant impact on debt financing and innovation performance. This shows that the improvement of corporate innovation performance is the common result of CEO narcissism and the increase of debt financing. CEOs with narcissistic characteristics can choose debt financing to alleviate the problem of insufficient investment capital of enterprises, optimize the allocation of resources invested, and ensure the improvement of transformative efficiency of R&D investment in all aspects. Therefore, debt financing will improve the innovation performance of narcissistic CEOs through the role of bridges and intermediaries.

The research conclusions provide a new behavioral perspective for corporate innovation motivation and enrich the research on the economic consequences of CEO narcissism. This paper expands the analytical framework of the relationship between CEO narcissism and corporate innovation performance by introducing corporate debt financing. This provides a reference for enterprises to obtain debt financing, for creditors to make loan decisions and some reference ideas and a theoretical basis for future research. Through empirical research, this paper finds that CEO narcissism is of great significance to enterprise innovation and corporate debt financing behavior. It provides more specific guidelines, mainly reflected in the following: it provides effective reference value for enterprises to appoint executives, provides more effective guidance in practice, and provides effective ideas for China to realize the development of its innovative companies. For an enterprise, an excellent CEO is reflected not only in his/her ability to deal with various affairs but also in his/her personality traits and self-awareness. Personal traits and psychological risk preferences should be matched with the company's image, positioning and overall strategy. Therefore, it is necessary to pay attention to the selection of CEOs, not only to examine their personal skills but also to integrate the CEO's own personality and psychological characteristics into the assessment system and pay attention to the level of narcissism, not only to avoid the negative impact of excessive narcissism on their personality and effective supervision of CEOs but also to pay attention to the positive role of narcissistic CEOs in enterprises. For creditors, attention should be given to the personal characteristics of the CEO of the debt enterprise to measure the CEO's ability to protect the creditor's rights and collect repayment from debtor companies.

Although some foreign studies have focused on leader narcissism as a starting point to study R&D investment behavior, China's economic, social and cultural environment is not the same as that of Western countries, so whether foreign scholars' conclusions can be further confirmed in China remains to be further explored. In the background of economic transformation and supply-side structural reform in contemporary China, China's enterprise innovation should not only focus on innovation input but also pay attention to performance output to avoid ineffective allocation and waste of resources. By comparing the differences between state-owned and non-state-owned enterprises in R&D investment, it is found that state-owned enterprises are characterized by high R&D investment and low innovation efficiency. It can be seen that state-owned enterprises have obvious advantages in innovation input, but what needs to be solved urgently is how to solve the problem of low innovation efficiency and alleviate the principal-agent conflict. Debt financing can provide financial support for enterprises' production, investment, innovation and research and development activities, and affect enterprises' innovation decisions and efficiency. The conclusion of this paper will help optimize the debt structure of state-owned enterprises and promote the reform of state-owned enterprises from the perspective of innovation-driven development. One is that Chinese listed companies should improve their articles of association and increase the terms of supervision over CEOs. The second is that enterprises should do a better job of separation of power and responsibility, improve corporate governance, and pay attention to enterprise R&D innovation. However, the decision-making mechanism should avoid the irrational investment and financing behavior of narcissistic CEOs caused by excessive concentration of power, and a benign balance between CEOs and executive teams should be formed. Third, we can consider granting certain benefits to the CEO for the achievement of results in which the CEO is consistent with the interests of the enterprise. Fourth, enterprises should strengthen internal audits, follow-up attention to corporate debt financing, timely correction of deviations, and control of corporate financial risks. Fifth, to create a healthy growth environment for narcissistic CEOs, the government should promote a good atmosphere of innovation and development, and strengthen the guidance of narcissistic CEOs in terms of the long-term interests of enterprise-oriented decision-making.

## Research Limitations

There may be some deficiencies in this study. First, since narcissism is an extremely complex psychological and personality feature, the objective indicators collected manually in this paper have certain errors, which cannot cover all the characteristics of narcissism and can only partially reflect some aspects of the narcissistic personality. Second, this paper mainly discusses the influence mechanism between CEO narcissism and enterprise innovation performance from the perspective of CEOs' own characteristics and debt financing. However, due to the limitations of the research, there may be other influencing mechanisms, and the influence of CEO narcissism on enterprise innovation is not fully investigated. To better clarify the impact of CEO narcissism on corporate innovation behavior, more boundaries, factors and paths can be considered to enrich the existing theoretical results. Since CEO narcissism is a complex personality trait, it is difficult to obtain the relevant data. The measurement data are mainly collected and collated manually, which may have some errors and need further improvement. This paper only explores the impact of CEO narcissism on enterprise innovation performance.

## Data Availability Statement

The original contributions presented in the study are included in the article, further inquiries can be directed to the corresponding author.

## Ethics Statement

Ethical review and approval was not required for the study on human participants in accordance with the local legislation and institutional requirements. Written informed consent for participation was not required for this study in accordance with the national legislation and the institutional requirements.

## Author Contributions

LZ and BL: writing. DB: providing revised advice. YZ and XY: processing data. All authors contributed to the article and approved the submitted version.

## Funding

This work was supported by Science and Technology Development Program of Jilin Province(China): Research on Problems and Countermeasures of Jilin Province's Economic Development Driven by Innovation of Science and Technology Service System; Doctoral Foundation of Jilin University of Finance and Economics: Research on the relationship between CEO power and firm growth of Gem listed companies in China.

## Conflict of Interest

The authors declare that the research was conducted in the absence of any commercial or financial relationships that could be construed as a potential conflict of interest.

## Publisher's Note

All claims expressed in this article are solely those of the authors and do not necessarily represent those of their affiliated organizations, or those of the publisher, the editors and the reviewers. Any product that may be evaluated in this article, or claim that may be made by its manufacturer, is not guaranteed or endorsed by the publisher.

## Footnotes

^1^The source of the CEO's signature is the IPO prospectus and offering prospectus of the listed company. The reason is that on May 17, 2006, the 180th Chairman's Office Meeting of China Securities Regulatory Commission approved the Measures for the Administration of Initial Public Offering and Listing. Article 43 of the Measures stipulates that the issuer and all of its directors, supervisors and senior managers shall sign and seal the prospectus/offering prospectus to ensure that the contents of the prospectus are true, accurate and complete. As the chairman of the sponsor or general manager to bear the corresponding legal responsibility, there will be no high-definition pictures affixed with the signature. The prospectus and the prospectus have a unified format, which facilitates the unified quantification of the signature size of the CEO in this paper.

^2^This paper takes the signature part of the prospectus as the main data source and makes a detailed comparison of the overlap with the CEO's signature in the prospectus and finds that the signature size of the two is basically the same. For this reason, the part about overlap is the prospectus.

## References

[B1] Alicke M. D. (1985). Global self-evaluation as determined by the desirability and controllability of trait adjectives. J. Pers. Soc. Psychol. 49:1621. 10.1037/0022-3514.49.6.1621

[B2] Baron R Kenny D. (1986). The moderator-mediator variable distinction in social psychological research: conceptual, strategic and statistical consideration. J. Pers. Soc. Psychol. 51, 1173–1182. 10.1037/0022-3514.51.6.1173 3806354

[B3] Bernile G. Bhagwat V. Rau P. R. (2017). What doesn't kill you will only make you more risk-loving: early-life disasters and CEO behavior. J. Financ. 72, 167–206. 10.1111/jofi.12432

[B4] Buyl T. Boone C. (2017). CEO narcissism, risk-taking, and resilience: an empirical analysis in U.S. commercial banks. J. Manage. 45:7699521 10.1177/0149206317699521

[B5] Campbell W. K. Goodie A. S. Foster J. D. (2004). Narcissism, confidence, and risk attitude. J. Behav. Decis. Mak. 17, 297–311. 10.1002/bdm.475

[B6] Campbell W. K. Hoffman B. J. Campbell S. M. (2011). Narcissism in organizational contexts. Hum. Resourc. Manage. Rev. 21, 268–284. 10.1016/j.hrmr.2010.10.007

[B7] Chatterjee A. Hambrick D. C. (2007). It's all about me: narcissistic chief executive officers and their effects on company strategy and performance. Adm. Sci. Q. 52, 351–386. 10.2189/asqu.52.3.351

[B8] Chatterjee A. Hambrick D. C. (2011). Executive personality, capability cues, and risk taking: how narcissistic CEOs react to their successes and stumbles. Adm. Sci. Q. 56, 202–237. 10.1177/0001839211427534

[B9] Chatterjee A. Poolock T. (2017). Master of puppets: how narcissistic CEOs construct their professional worlds. Acad. Manage. Rev. 42, 703–725. 10.5465/amr.2015.0224

[B10] Choi S. B. Lee S. H. Williams C. (2011). Ownership and firm innovation in a transition economy: evidence from China. Res. Policy. 40, 441–452. 10.1016/j.respol.2011.01.004

[B11] David P. O'Brien JP. Yosikawa T. (2008). The implications of debt heterogeneity for RandD investment and firm performance. Acad. Manage. J. 51, 165–181. 10.5465/amj.2008.30772877

[B12] Demerjian P. Lev B. Mcvay S. (2012). Quantifying managerial ability. Manage. Sci. 3, 1229–1248. 10.1287/mnsc.1110.1487

[B13] Emmons R. A. Robert A. (1987). Narcissism: theory and measurement. J. Pers. Soc. Psychol. 52, 11–17. 10.1037/0022-3514.52.1.11 3820065

[B14] Freud S. (1915). On narcissism: an introduction, in The Standard Edition of the Complete Works of Sigmund Freud (Vol. XIV). London: Hogarth Press.

[B15] Galasso A. Simcoe T.S. (2011). CEO overconfidence and innovation. Manage. Sci. 57, 1469–1484. 10.1287/mnsc.1110.1374

[B16] Gerstner W. C. Koenig A. Enders A. Hambrick D. C. (2013). CEO narcissism, audience engagement, and organizational adoption of technological discontinuities. Adm. Sci. Q. 58, 257–291. 10.1177/0001839213488773

[B17] Gervais S. Heaton J.B. O dean T. (2011). Overconfidence, compensation contracts, and capital budgeting. J. Financ. 66, 1735–1777. 10.1111/j.1540-6261.2011.01686.x

[B18] Hackbarth D. (2008). Managerial traits and capital structure decisions. J. Financ. Quant. Anal. 43, 843–882. 10.1017/S002210900001437X

[B19] Ham C. Lang M. Seybert N. Wang S. (2017). CFO narcissism and financial reporting quality. J. Account. Res. 55, 1089–1135. 10.1111/1475-679X.12176

[B20] Ham C. Seybert N. Wang S. (2018). Narcissism is a bad sign: CEO signature size, investment, and performance. Rev. Account. Stud. 23, 1–31. 10.1007/s11142-017-9427-x

[B21] Hambrick D.C. (2007). Upper echelons theory: an update. Acad. Manage. Rev. 32, 334–343. 10.5465/amr.2007.24345254

[B22] Hambrick D.C. Mason P.A. (1984). Upper echelons: the organization as a reflection of its top managers. Acad. Manage. Rev. 9, 193–207. 10.5465/amr.1984.4277628

[B23] Heaton J. B. (2002). Managerial optimism and corporate finance. Financ. Manage. 31, 33–45. 3666221 10.2307/3666221

[B24] Hirshleifer D. Low A. Teoh S. H. (2012). Are overconfident CEOs better innovators? J. Financ. 67, 1457–1498. 10.1111/j.1540-6261.2012.01753.x

[B25] Kaplan S. Klebanov M. Sorensen M. (2012). Which CEO characteristics and abilities matter? NBER Work. Pap. 67, 973–1003. 10.1111/j.1540-6261.2012.01739.x

[B26] Kim H. Lee K. (2008). Corporate governance ownership structure and the relationship between financial slack and RandD investments: evidence from Korean firms. Organiz. Sci. 19, 404–418. 10.1287/orsc.1080.0360

[B27] Kumar V, D. (2019). Multiple faces of narcissistic leadership in medical education. J. Adv. Med. Educ. Prof. 7, 103–105. 10.30476/JAMP.2019.44705 31086802 PMC6475033

[B28] Larwood L. Whittaker W. (1977). Managerial myopia: self-serving biases in organizational planning. J. Appl. Psychol. 62, 194–198. 10.1037/0021-9010.62.2.194

[B29] Lee J. M. Hwang B. H. Chen H. (2016). Are founder CEOs more overconfident than professional CEOs? evidence from SandP 1500 companies. Strateg. Manage. J. 38, 751–769. 10.1002/smj.2519

[B30] Lu Z. Zhu J. Zhang W. (2012). Bank discrimination, holding bank ownership, and economic consequences: evidence from china. J. Bank. Financ. 36, 341–354. 10.1016/j.jbankfin.2011.07.012

[B31] Malmendier U. Tate G. (2005). CEO overconfidence and corporate investment. Finance 60, 2661–2700. 10.1111/j.1540-6261.2005.00813.x

[B32] Miller D. Ross M. (1975). Self-serving biases in the attribution of causality: fact or fiction? Psychol. Bull. 82, 213–225. 10.1037/h0076486

[B33] Olsen K. J Stekelberg J. (2016). CEO narcissism and corporate tax sheltering. The J. Am. Luxat. Assoc. 38, 1–22. 10.2308/atax-51251

[B34] Olsen K. J. Dworkis K. K. Young S. M. (2014). CEO narcissism and accounting: a picture of profits. J. Manage. Account. Res. 26:2. 10.2308/jmar-50638

[B35] Rosenthal S. A. Pittinsky T. L. (2006). Narcissistic leadership. Leader. Q. 17, 617–633. 10.1016/j.leaqua.2006.10.005

[B36] Shefer D. Frenkel A. (2005). RandD, firm size and innovation: an empirical analysis. Technovation 25, 25–32. 10.1016/S0166-4972(03)00152-4

[B37] Shefrin H. Statman M. (1999). Making sense of beta, size and book-to-market: investor expectations are consistent–but wrong. J. Portfolio Manage. 21, 26–34. 10.3905/jpm.1995.409506

[B38] Steen E. (2005). Organizational beliefs and managerial vision. Econ. Organ. 21, 256–283. 10.1093/jleo/ewi011

[B39] Sunder J. Sunder S. V. Zhang J. (2017). Pilot CEOs and corporate innovation. J. Financ. Econ. 123, 209–224. 10.1016/j.jfineco.2016.11.002

[B40] Thakor A. V. Goel A. M. (2008). Overconfidence, CEO selection, and corporate governance. J. Financ. 63, 2737–2784. 10.1111/j.1540-6261.2008.01412.x

[B41] Wallace H. Baumeister R. (2002). The performance of narcissists rises and falls with perceived opportunity for glory. J. Pers. Soc. Psychol. 82, 819–834. 10.1037/0022-3514.82.5.819 12003480

[B42] Wang C. Kafouros M I. (2009). What factors determine innovation performance in emerging economies? Int. Bus. Rev. 18, 606–616. 10.1016/j.ibusrev.2009.07.009

